# Spatial Reorientation by Geometry in Bumblebees

**DOI:** 10.1371/journal.pone.0037449

**Published:** 2012-05-18

**Authors:** Valeria Anna Sovrano, Elisa Rigosi, Giorgio Vallortigara

**Affiliations:** 1 Centre for Mind/Brain Sciences, University of Trento, Rovereto, Italy; 2 Departments Sustainable Agro-ecosystems and Bioresources, Research and Innovation Centre, Instituto Agrario Di San Michele All'Adige (IASMA), Fondazione Edmund Mach, San Michele all'Adige, Italy; Royal Holloway University of London, United Kingdom

## Abstract

Human and non-human animals are capable of using basic geometric information to reorient in an environment. Geometric information includes metric properties associated with spatial surfaces (e.g., short *vs.* long wall) and left-right directionality or ‘sense’ (e.g. a long wall to the left of a short wall). However, it remains unclear whether geometric information is encoded by explicitly computing the layout of surface geometry or by matching images of the environment. View-based spatial encoding is generally thought to hold for insect navigation and, very recently, evidence for navigation by geometry has been reported in ants but only in a condition which does not allow the animals to use features located far from the goal. In this study we tested the spatial reorientation abilities of bumblebees (*Bombus terrestris*). After spatial disorientation, by passive rotation both clockwise and anticlockwise, bumblebees had to find one of the four exit holes located in the corners of a rectangular enclosure. Bumblebees systematically confused geometrically equivalent exit corners (i.e. corners with the same geometric arrangement of metric properties and sense, for example a short wall to the left of a long wall). However, when one wall of the enclosure was a different colour, bumblebees appeared to combine this featural information (either near or far from the goal) with geometric information to find the correct exit corner. Our results show that bumblebees are able to use both geometric and featural information to reorient themselves, even when features are located far from the goal.

## Introduction

Following the seminal work by Cheng [1] a variety of vertebrate species have been shown to be able to reorient in space using geometric cues, i.e. using *metric properties* of surfaces, such as choosing between a short wall and a long wall, and *sense discrimination*, such as choosing between turning to the wall located to left or right, including fish: [2]–[5]; domestic chicks: [6]–[9]; pigeons: [10], [11]; rhesus monkeys: [12]; rats: [13], [14]; human children: [15]–[20]. In these studies, animals are first allowed to locate a hidden goal at one of the corners of a rectangular enclosure, in the absence of extra-enclosure cues (landmarks). They are then disoriented (passively) by slow turning in the dark into a small container outside the apparatus (by several complete 180 degree turns), and finally re-introduced to the rectangular enclosure and allowed to reorient and search for the goal. Typically animals direct their searches towards the correct corner and its geometric equivalent, namely the corner which is located diagonally opposite with respect to the goal. These two corners are similar with respect to the metric and sense relationships relative to the enclosure's walls (for example, both are characterized by a long wall to the right of a short wall), and are therefore geometrically indistinguishable. When nongeometric (featural) information is added to the rectangular enclosure, for instance a differently coloured wall or a conspicuously different panel located at the corner, animals appear to be able to combine geometric and featural information to distinguish between geometrically equivalent corners. Reliance on geometric (rather than featural) information is more pronounced in small enclosures [21], [22], but in general use of geometry appears to be almost universal [23] and observed at birth in vertebrates [24]–[27].

Recently, evidence that invertebrates can also use geometry in the rectangular enclosure task has been reported for the first time in the neotropical ant *Gigantiops destructor* [28]. This is important from a comparative and evolutionary perspective, but also from a theoretical point of view. It is generally agreed that insects navigate using view-based homing strategies [29]–[31], such that insects would navigate to minimize the difference between the memorised panoramic image of the goal site and the panorama perceived from the current location [32]. Theoretical and computer modelling suggest that view-based strategies for reorientation could in principle produce rotational (geometric) errors in the rectangular enclosure task [33]–[38] and therefore may also be used by vertebrates. Recent evidence to support this view has been obtained from domestic chicks, although rather than using a rectangular enclosure this test used a rectangular array of freestanding objects [39].

The aim of this paper is twofold. Firstly, we wanted to investigate the ability of a group of insects that have not yet been tested, Apidae - and that is well-known for its navigational and cognitive abilities - to reorient by geometry using bumblebees (*Bombus terrestris*). Though less studied than honeybees [40], bumblebees have also been shown to be capable of different cognitive feats (e.g., [41]), including complex routing problems when foraging (analogous to the Travelling Salesman Problem; [42], [43], [44]) and also estimation of distances based on the number of landmarks [45]–[47]. Secondly, we wanted to investigate bumblebees ability to reorient when featural information is located near or far from the goal. Wystrach and Beugnon [28] studied ants using panels located at the corners of a rectangular arena as featural information, which did not allow them to investigate the use of features located far from the goal. Instead we used the traditional task employed with vertebrate species in which one wall of the enclosure is a different colour and may be located on the same wall with respect to the goal corner (thus acting as a beacon that provides a direct sensory cue to the goal) or on different wall with respect to the goal corner (thus acting as a true landmark). We expected bumblebees to be able to use featural information even when located on a different wall with respect to the goal corner.

## Experiment 1

In the first experiment bumblebees were tested for reorientation when only geometric cues were available, i.e. in a rectangular enclosure in which only the length of the enclosure walls (metric differences) and their relative position (e.g. long wall to the right of a short wall), but not featural information, could be used for reorientation. The design of the test is similar to that used with most vertebrate species, and employed a reference memory paradigm.

### Methods

#### Subjects

*Bombus terrestris* colonies were supplied by Bioplanet s.c.a. (Cesena, Italy). They came without previous foraging experience, and were reared in our laboratories (temperature: 25°C, natural illumination). Animals were fed daily with pellets of fresh pollen and water mixed with honey. Adult foragers (mean body length: 1.7 cm; mean thorax width: 0.7 cm) were used from two different colonies for the two experiments [47], [48], [49]. Ten bumblebees were used in Exp. 1 and twenty in Exp. 2.

#### Apparatus

**Figure 1:**
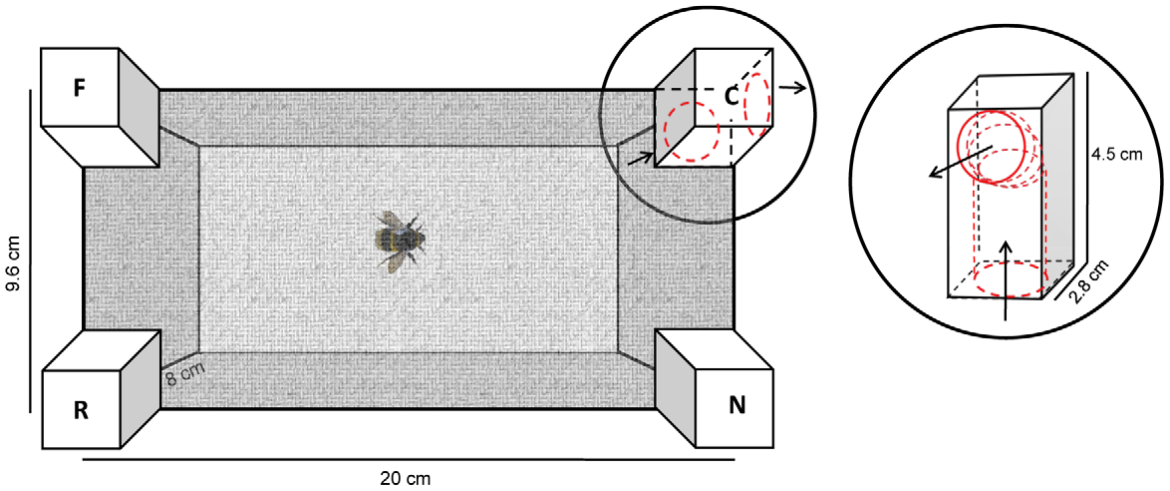
Schematic view of the test apparatus. Corners are conventionally identified as C = Correct, R = Rotation (geometrically equivalent corner), F = Far, N = Near. Only the external hole of the correct corner was opened allowing bumblebees to exit.

The experimental set up consisted of a rectangular enclosure (Fig. 1) of green plastic (Poliplak ®), 20 cm long and 9.6 cm wide, with 8-cm-high walls. The internal walls of the enclosure were lined with replaceable thin green cardboard. The enclosure was covered on top with a rectangular insect net (21 cm×10.2 cm×3 cm). In each corner an L-shaped wooden block (2.8×2.8 cm at the base, and 4.5 cm high) was inserted through a hole in the net (see detail in Fig. 1). An opening in the block gave access from the inside of the enclosure to an L-shaped corridor (1.5 cm in diameter) through which a bee could pass (the exit of which was not visible from the entrance because of the L-shaped structure of the block). All external exits from the blocks were closed by nets except for one (positive or reinforced) that allowed the animal to leave the enclosure. The testing enclosure was inserted in a larger polyester and vinyl insect rearing tent (60 cm×60 cm×60 cm; Mega View Science Co., Ltd, Taiwan) where food (fresh pollen) was randomly located in 6–8 spots on the floor, thus providing motivation to the animals to exit the rectangular enclosure in repeated trials. All experiments were video-recorded with a video camera (Sony Handycam dcr-sr87), positioned 20 cm above the tent using a tripod.

#### Procedure

Bumblebees were given 2 training sessions per day, each consisting of 8 trials with a 40 minutes inter-session interval. During each trial the bumblebee was placed in the centre of the arena using a small opaque container (5.5 cm in diameter and 7 cm high) and the number of attempts to exit from the blocks located in the four corners was recorded until the bumblebee was able to locate the correct exit and fly into the larger tent. A bumblebee was considered to have made a choice when its entire body had gone through the hole in one of the corner blocks (this was checked by direct inspection during the experiments and recorded in the sound track accompanying the video recording for subsequent analyses). In each trial, the maximum time allowed to exit the rectangular enclosure was 20 min, after which the animal was disoriented and given another trial (the disorientation procedure involved placing the animal in a closed, opaque small container, 5.5 cm in diameter and 7 cm high, and gently rotating it 360° both clockwise and anticlockwise several times). When the animal chose the correct corner at the first attempt it was allowed a 10 minutes period of reinforcement (during which the bumblebee was free to fly and feed in the larger tent); when the animal was able to chose the correct corner and exit only after attempts at the other corners it was given a shorter period of reinforcement in the larger tent (3 min). From trial to trial the rectangular enclosure was rotated 90° clockwise, in order to prevent use of external cues, and, before any trial, the bumblebee underwent the passive disorientation treatment. After the disorientation procedure, the bumblebee was reintroduced to the rectangular enclosure for the subsequent trial. During the inter-session interval the bumblebees were kept individually in the opaque container to allow identification.

#### Data analyses

The number of times each bee attempted to exit the enclosure at for each corner were computed for all eight trials (attempts refer to entering a wood block, including the correct one C, with the entire body without exiting). We also considered for each bumblebee the corner chosen first in each of the eight trials. These behavioral measurements were similar to those used in the same type of experiments with vertebrates (see for details of methods [2]–[5], [10]). These data were entered into an analysis of variance (ANOVA) with sessions and corners as within subject factors. Corners were identified as follows: C = Correct, R = Rotation (geometrically equivalent corner), F = Far, N = Near.

### Results

**Figure 2:**
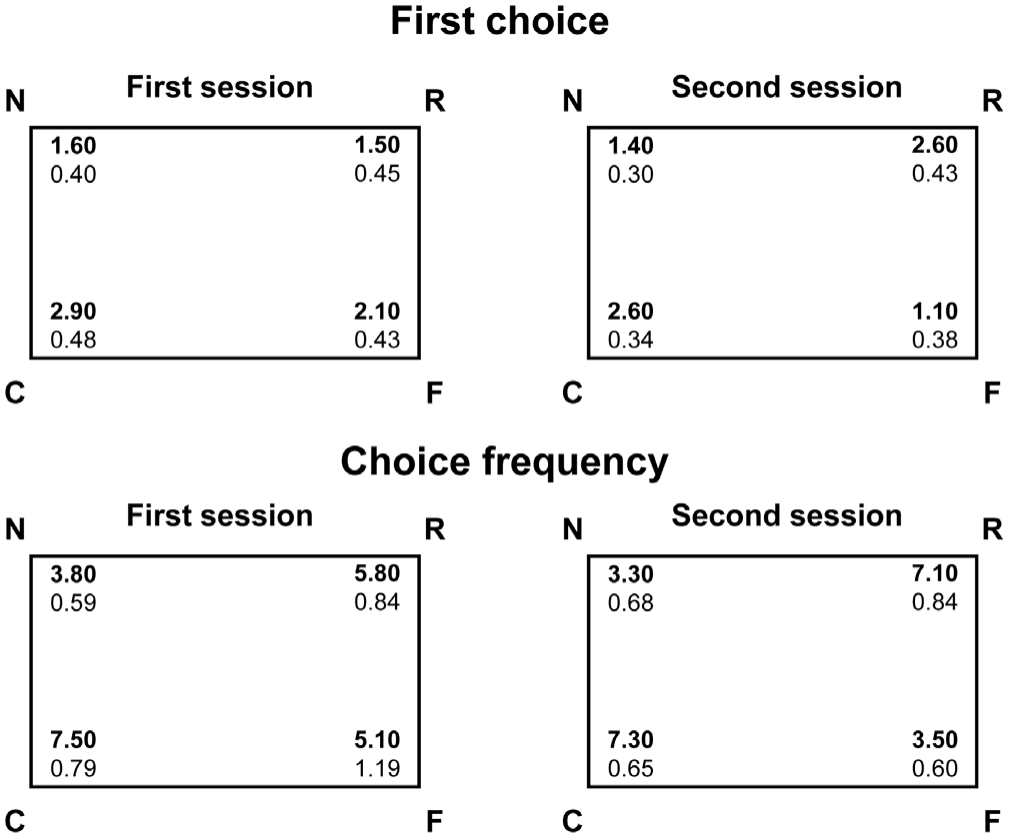
Top panels: the mean number of first choices made to each corner by the group of bumblebees (Exp. 1; groups means with SEM are shown) per session. Bottom panels: the mean number of times (choice frequency) the same bees (group means with SEM are shown) visited each corner per session.

Analyses of first choices (Fig. 2 top) revealed that bumblebees did not choose at random between the four corners but showed a systematic choice for certain corners in both sessions (the general ANOVA revealed a significant main effects of corners (C,R,N,F; (F(3,27) = 3.446 p = 0.031).

In particular, when comparing choices for the two geometrically correct corners (C, R) versus the two geometrically incorrect corners (N, F) we observed a significant difference (F(1,9) = 7.741 p = 0.021), with more choices for corners C,R (this is clear from the top panel in session 2, but not session 1 (as expected), though the ANOVA failed to reveal a significant corners (CR *vs.* NF)×sessions (first *vs.* second) interaction (F(1,9) = 1.714 p = 0.223)). Thus, bumblebees chose the two geometrically correct corners (C,R) over the two geometrically incorrect corners (N,F), and did so from the first session of trials.

No significant difference was apparent in first choice frequency between the correct corner, C, and its geometrically equivalent corner R (F(1,9) = 1.569 P = 0.242), indicating that the disorientation procedure was effective and that bumblebees did not rely on any extra- or intra-enclosure featural cues.

Analyses on frequency of choices (Fig. 2 bottom) confirmed that bumblebees choices were not equally distributed among the four corners (the general ANOVA revealed only a significant main effect of corners (C,R,N,F; F(3,27) = 12.594 p<0.001).

When comparing the choice frequency in the two geometrically correct (C, R) versus the two geometrically incorrect corners (N, F) a highly significant difference was apparent (F(1,9) = 61.714 p<0.0001), revealing more choices for the two geometrically correct corners (C,R).

No significant difference was apparent in choice frequency between the correct corner, C, and its geometrically equivalent, corner R (F(1,9) = 0.819 P = 0.389), indicating that the disorientation procedure was effective and that bumblebees did not rely on any extra- or intra-enclosure featural cues.

Analyses of both first choices and choice frequencies thus clearly support the view that bumblebees reoriented on the basis of geometric information.

## Experiment 2

In the second experiment we tested the ability of bumblebees to make use of featural cues in order to distinguish between the two geometrically equivalent corners in the rectangular enclosure escape task. As in similar experiments carried out with vertebrates, one of the walls of the enclosure was a different colour, to provide the animals with information to allow discrimination between geometrically equivalent corners.

### Methods

#### Apparatus, Procedure and Data Analyses

**Figure 3:**
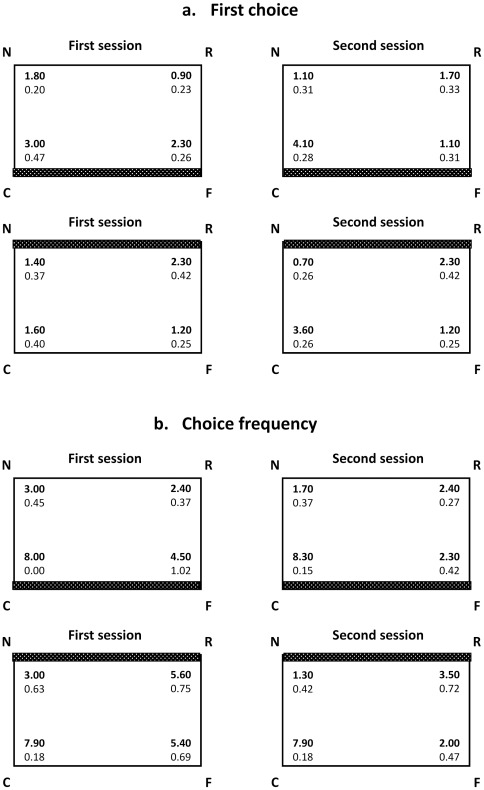
(A) The mean number of first choices made to each corner by the group of bumblebees (Exp. 2; groups means with SEM are shown) per session. Top panels: Near-feature: the feature (white wall indicated by the horizontal grey stripe, all other walls are green) is located at the target corner C; Bottom panels: Far-feature: the feature (white wall indicated by the horizontal grey stripe, all other walls are green) is located away from the target corner C. (B) The mean number of times (choice frequency) bumblebees visited each corner (Exp. 2; groups means with SEM are shown) per session. Top panels: Near-feature: the feature (white wall indicated by the horizontal grey stripe, all other walls are green) is located at the target corner C; Bottom panels: Far-feature: the feature (white wall indicated by the horizontal grey stripe, all other walls are green) is located away from the target corner C.

**Figure 4:**
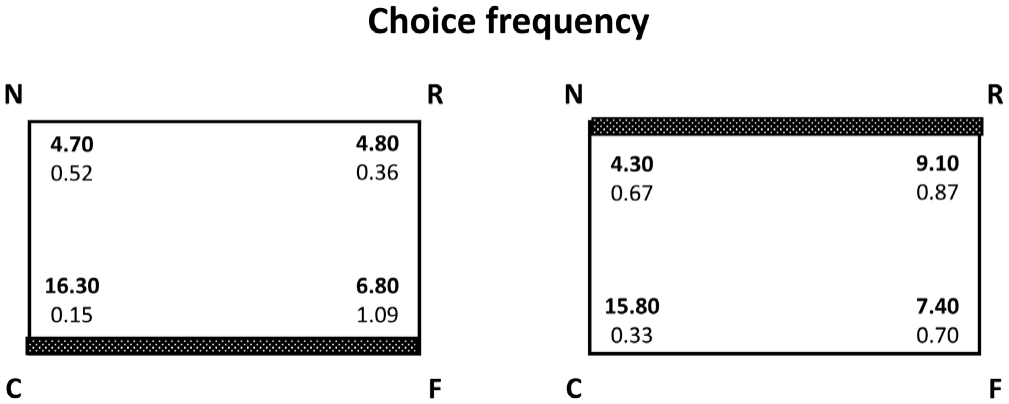
The mean number of times (choice frequency) bumblebees visited each corner (group means with SEM are shown). Data for the two testing sessions were pooled because there was no significant interaction in the general ANOVA between distance from the feature and session (Exp. 2). Left panel: Near-feature: the feature (white wall indicated by the horizontal grey stripe, all other walls are green) is located at the target corner C. Right panel: Far-feature: the feature (white wall indicated by the horizontal grey stripe, all other walls are green) is located away from the target corner C.

The apparatus was the same as in the previous experiment; this time, however, one of the longer walls was covered with white cardboard. For half of the animals (N = 10) the feature (the white wall) was located near the correct corner (i.e. bumblebees were trained with a green-white corner as positive, see Fig. 3 and 4). For the other half (N = 10) the feature was located far from the correct corner (i.e. bumblebees were trained with a green-green corner as positive, see Fig. 3 and 4). The data were analyzed by ANOVA with corners (C,R,N,F) and sessions (first, second) as a within-subjects factors, and distance from feature (near-feature, far-feature) as a between-subjects factor.

### Results

Analyses of first choices (Fig. 3a) showed that the distribution of bumblebee choices among the four corners was dependent on both distance from feature and the (testing) session (the general Anova revealed only significant main effects of corners, F(3,54) = 15.478 p<0.001, and distance from the feature×corners, F(3,4) = 4.445 p = 0.007, and sessions×corners, F(3,54) = 12.180 p<0.001, interactions).

In particular, when the correct corner was near the feature (animals trained on the corner with differently colored walls as positive) bumblebees could discriminate C from R (F(1,9) = 12.062 p = 0.001) but they still appeared to confuse the two corners with similar features (C vs. F: F(1,9) = 0.957) in the first session. However, in the second session bumblebees chose the correct corner first significantly more often than all other corners (there was a significant difference when comparing all four corners C,R,F,N: F(3,27) = 15.826 p = 0.001, but not when comparing only corners N,R,F: F(2,18) = 0.844).

When the correct corner was far from the feature (animals trained on the corner with identically coloured walls as positive) no significant effect of corner was observed in the first session (F(3,27) = 1.561 p = 0.221). In the second session most choices were made to the correct corner (C), and although there was some evidence for rotational errors (choices made to corner R), bees first chose corner C significantly more than R (F(1,9) = 5.444 p = 0.042).

Analyses confirmed that bumblebees discriminated between the four corners (Fig. 3b) and that choice frequency was affected by both session and distance from feature (the general ANOVA revealed only significant main effect of corner, F(3,54) = 172.041 p<0.0001, session, F(1,54) = 15.087 p = 0.001, and significant corners×distance from feature, F(3,54) = 8.494 p<0.001, and sessions×corners, F(3,54) = 5.571 p = 0.002, interactions).

When the correct corner was near the feature (animals trained on the corner with differently coloured walls as positive) there was a marginally significant difference in choice frequency between corners R, N and F (F(2,18) = 3.718 p = 0.044) with only a trend for a significant interaction with sessions (F(2,18) = 2.865 p = 0.083). As can be seen from Fig. 4, in which data for the two sessions were pooled because there was no significant interaction in the general ANOVA between distance from the feature and sessions, this difference was mainly due to a slightly higher number of errors in the F corner, the corner sharing the featural cue with the corner C (Fig. 4). No evidence for geometric errors was apparent (choice for N and R were similar, see leftmost panel in Fig. 4).

When the correct corner was far from the feature (animals trained on the corner with identically-colored walls as positive) there was a difference in choice frequency between corners R, N and F (F(2,18) = 20.961 p<0.0001; no significant interaction with sessions was observed (F(2,18) = 0.934 p = 0.411). This was due to both errors in the F corner (sharing the same featural characteristic as corner C) and geometric errors (choosing corner R: Fig. 4).

There were two main results. First, bumblebees appeared to be able to combine geometric and featural information to distinguish between geometrically equivalent locations: bumblebees searched mostly in the correct corner C after some training (i.e. in the second session), irrespective of the visual characteristics of the corner (with identically-coloured or differently-coloured walls). Second, the distribution of errors, particularly for choice frequency, was different depending on the distance from the feature to the goal: bumblebees trained to a corner near to the feature (i.e. made of differently-coloured walls) committed mostly errors based on similarity of features (at least during first session in first choice), i.e. choices for corner F rather than corners N and R, whereas bumblebees trained to a corner far from the feature (i.e. made of identically-coloured walls) committed both errors based on similarity of features and errors based on similarity of geometry, i.e. choices for both corner F and corner R. Errors involving visits to the corner that share the same local featural appearance (F) seem to be similar in the two conditions, what is different is the number of geometric errors (choices for R), which was higher in the condition in which bumblebees were trained to the corner with the two similar walls as positive (i.e., with the feature far from the goal). In this case, bumblebees visited the R corner in spite of its being marked by a wall of a different colour.

### Discussion

The results show that under similar conditions of testing, bumblebees exhibit the same behaviour during spatial reorientation as shown by vertebrates. They appear to be able to reorient themselves using purely geometric cues (Exp. 1), as revealed by the pattern of confusion between geometrically equivalent locations (C and R), i.e. the fact that they chose the correct corner (C) and its geometrically equivalent corner (R) with similar frequency. Moreover, bumblebees also distinguish between geometric equivalent locations (C and R) when tested in the presence of featural information, both near and far from the goal (Exp. 2). This confirms the results obtained with ants [28], [50], [52] and also extends the findings because ants were tested with panels located at the corners as featural information, and therefore there was no evidence that they could use features for reorientation which were located far from the goal (i.e., features not located in the correct corner; but see [50] for more recent evidence).

As to the mechanisms bumblebees may use for reorientation, it is commonly believed that insects navigation is based on a view-based (‘snapshot’) matching strategy ([31]; see however [53] for a view stressing allocentric rather than view-centred mechanisms). In order to relocate a goal, insects would rely on a memorized view taken at the goal location; moving in the environment they would compare their current view with the memorized view of the goal, proceeding from high to low levels of mismatch until the views are perfectly matched. Recent computational evidence [35], [36] suggests that such a global image matching gradient-descent strategy may hold in principle also for reorientation in a rectangular environment. With a view-image taken at the target corner, the visual weight of its global shape of the rectangular enclosure would overcome local characteristics located at the corners, creating local minima in mismatch at both target location and its geometrically equivalent location, thus producing rotational errors. It has been also suggested that image-matching processes at two different spatial scales would capture a combination of geometric and featural cues [28], [50], [52]: first, global matching on a large spatial scale would capture geometrically equivalent locations as local minima of a gradient-descent algorithm, and then considering the quality of the matching at a local minimum on a finer spatial scale making local features at the corners prominent.

The hypothesis is attractive because it suggests that encoding geometry would in fact be not an explicit process, i.e., that metric and sense would be only implicitly encoded by the global matching process on the basis of salient brightness contours in the image. Moreover, the distinction between the encoding of the geometry of space and the encoding of the features would vanish, because it would simply be the result of two successive processes of view-based matching [28], [51], [52]. Whether the hypothesis would hold for vertebrate reorientation is a matter for debate, because there is evidence for dissociable neural mechanisms underlying the encoding of geometry and featural information in vertebrates [7], [54], [55] and some recent evidence in children [56] and chicks [57] supporting the view that they use indeed the layout of the surface geometry in the environment and not the matching images of brightness contours.

What about bumblebees? Does the evidence we obtained in the experiments support the hypothesis that they use a global matching gradient-descent algorithm? The overall evidence does support the view-matching hypothesis but some aspects of the results of Exp. 2 are puzzling. When trained with the feature near the goal, bumblebees made more errors in corner F than in corners N and R (see Fig. 4). Clearly they searched for the feature in spite of the fact that in corner F the feature was located to the right side when approaching that corner, whereas it was located to the left side when approaching the correct C corner - and this is something that should be easily detected by any view matching mechanism (see [58], [59] for evidence that visual memory of bees does not show a mirror-image ambiguity). The second puzzling result is that choices for R (rotational errors) were rare when bumblebees were trained with the feature near the goal and very common when trained with the feature far from the goal (Fig. 4). These results can perhaps be accounted for by assuming that, besides global matching, bumblebees use a separate process for reorientation which is based on the mere detection of the presence of the feature, irrespective of its location with respect to left-right sense. This could explain high error rates in corner F, because of attraction to the corner with a similar colour pattern to the training corner (C) would occur irrespective of the left/right positioning of the colours themselves. Moreover, it could also explain the pattern of rotational errors (choices for the R corner) in the near and far from the feature conditions. The presence of the feature would be encoded when bumblebees are trained in the near-feature condition, thus producing few rotational errors (because of lack of such a feature in corner R), whereas it would be not encoded when bumblebees are trained in the far from the feature condition, thus producing common rotational errors because the presence of the feature in corner R would be ignored and choices of bumblebees would be based on geometry alone.
